# Turbo‐Synergistic Oily Wastewater Remediation in Bio‐Inspired Cone Array Barrel

**DOI:** 10.1002/advs.202204244

**Published:** 2022-10-06

**Authors:** Bing Wang, Xianfeng Luo, Yawei Feng, Linfeng Yang, Chunhui Zhang, Zhichao Dong, Lei Jiang, Haoyu Dai

**Affiliations:** ^1^ CAS Key Laboratory of Bio‐inspired Materials and Interfacial Sciences Technical Institute of Physics and Chemistry Chinese Academy of Sciences Beijing 100190 China; ^2^ School of Future Technology University of Chinese Academy of Sciences Beijing 101407 China; ^3^ A123 Operation Systems Wanxiang A123 Systems Corp. Hangzhou 311215 China

**Keywords:** bio‐inspired structure gradient, emulsion separation, liquid dynamics, superwettability

## Abstract

Oily wastewater discharge causes not only the pollution of environment but also the waste of resources. Existing technologies for wastewater remediation, such as membrane and particle methods, are variable and effective, but are difficult for achieving continuous and rapid oil–water separation. Here, with the synergy of turbo stirring, a strategy for emulsion separation is demonstrated based on the bio‐inspired cone array barrel. Under the centrifugal force, oil droplets in emulsion are thrown onto the cones arrayed on inner wall due to the Coriolis effect, captured by microstructures on cone surface and then penetrate out through the superhydrophobic pores, while only the remediated water remains. The separation technique maintains a high efficiency of above 99.5% for over 30 times of use, as well as for emulsions with variable ingredients. This structure‐dynamics synergistic separation strategy evolves the future technologies on water purification in industrial and daily processes.

## Graphical abstract

A new strategy for wastewater remediation is developed based on the synergy of turbo stirring and gradient structure. Under centrifugal force, the oil droplets in emulsion can continuously be captured on inner wall and then penetrate out from the cone array barrel, in which remediated water remains. Future technologies of water purification can be predicted based on this work.

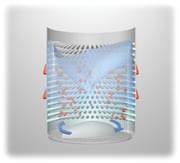

## 1 Introduction

Oily wastewater discharge from industrial, daily, and transport processes such as chemicals, domestic sewage, and marine oil leakage, will cause severe pollution to the ecological environment of oceans and excess waste of water or oil resources.^[^ ^1^, ^2^, ^3^ ^]^ External forces (ocean current movement, for instance) motivates the stratified oil–water phase to form a more stable emulsion, which makes a lasting impact on the environment, as well as rises the difficulty and cost of oil–water separation. Researchers mainly adopt relatively static methods, such as gravity separation, flotation, and membrane separation, for the separation of oil–water mixture and emulsion.^[^ ^4^, ^5^, ^6^, ^7^ ^]^ In the relatively static case, the mixture of oil and water is stratified by density difference.^[^ ^8^, ^9^, ^10^, ^11^ ^]^ As the liquid of continuous phase drains through the membrane, the droplets of dispersed phase gradually accumulate based on the size‐sieving effect, to contaminate or eventually block the pores, which will increase the separation difficulty.^[^ ^12^, ^13^, ^14^ ^]^ As for the dynamic case, particles are commonly added into the emulsion to contact and adsorb the oil droplets of dispersed phase by means of violent oscillation, and aggregate afterward to form the oil–water stratification.^[^ ^15^, ^16^ ^]^ However, it is difficult for particles in continuous separation due to the problems of secondary separation and recovery. Therefore, dynamic emulsion separation calls for a new strategy due to these inevitable limitations.

In nature, many creatures such as cactus,^[^ ^17^ ^]^ green bristlegrass,^[^ ^18^ ^]^ and the *Cotula fallax* plant^[^ ^19^ ^]^ have the ability to continuously collect water in the fog due to their anisotropic cone structure.^[^ ^20^, ^21^ ^]^ The differences in the radius of curvature between the front and the end of the droplet on the cone lead to the Laplace pressure, which contributes to the spontaneous transportation of droplet. Relevant capability is imitated to fabricate many bio‐inspired cone array devices for fog or bubble collection.^[^ ^22^, ^23^, ^24^, ^25^ ^]^ Micro‐nanostructured cone array tube^[^ ^26^ ^]^ has been designed in our previous work to achieve versatile and durable emulsion separation, still, facing the difficulty to separate emulsion in dynamic case. On the one hand, washing machine could remove the greasy dirt off the clothes by turbo stirring. On the other hand, continuous multistep chemical reactions could be achieved without too much manual involvement by using a high‐speed rotating container.^[^ ^27^ ^]^ Inspired by the synergy of gradient structure and centrifugal dynamics, we propose a strategy for oily wastewater remediation, attributing to the turbo‐synergistic bio‐inspired cone array barrel (CAB). Under the centrifugal force in CAB, oil droplets in emulsion are thrown onto the cones arrayed on inner wall due to the Coriolis effect, captured by microstructures on cone surface, and then penetrated out through the superhydrophobic pores, while only remediated water remains. The separation technique maintains a high efficiency of above 99.5% for over 30 times of use, as well as plays a good role in separating emulsions with variable ingredients (even with nonionic surfactant). Experimental results reveal that larger height, smaller spacing, and larger apex angle of cone contribute to higher separation efficiency. With attempts of separation under different rotation rates, turbo‐synergistic CAB in 1000 rpm has been decided to further use. Furthermore, a CAB evolved water purifier has been designed to effectively remediate the liquid flown in. Drawing up the blueprint of turbo‐synergistic CAB‐inspired wastewater plant, we anticipate that this strategy has the potential to predict future technologies in wastewater treatment.

## 2 Results and Discussion

**Figure 1:**
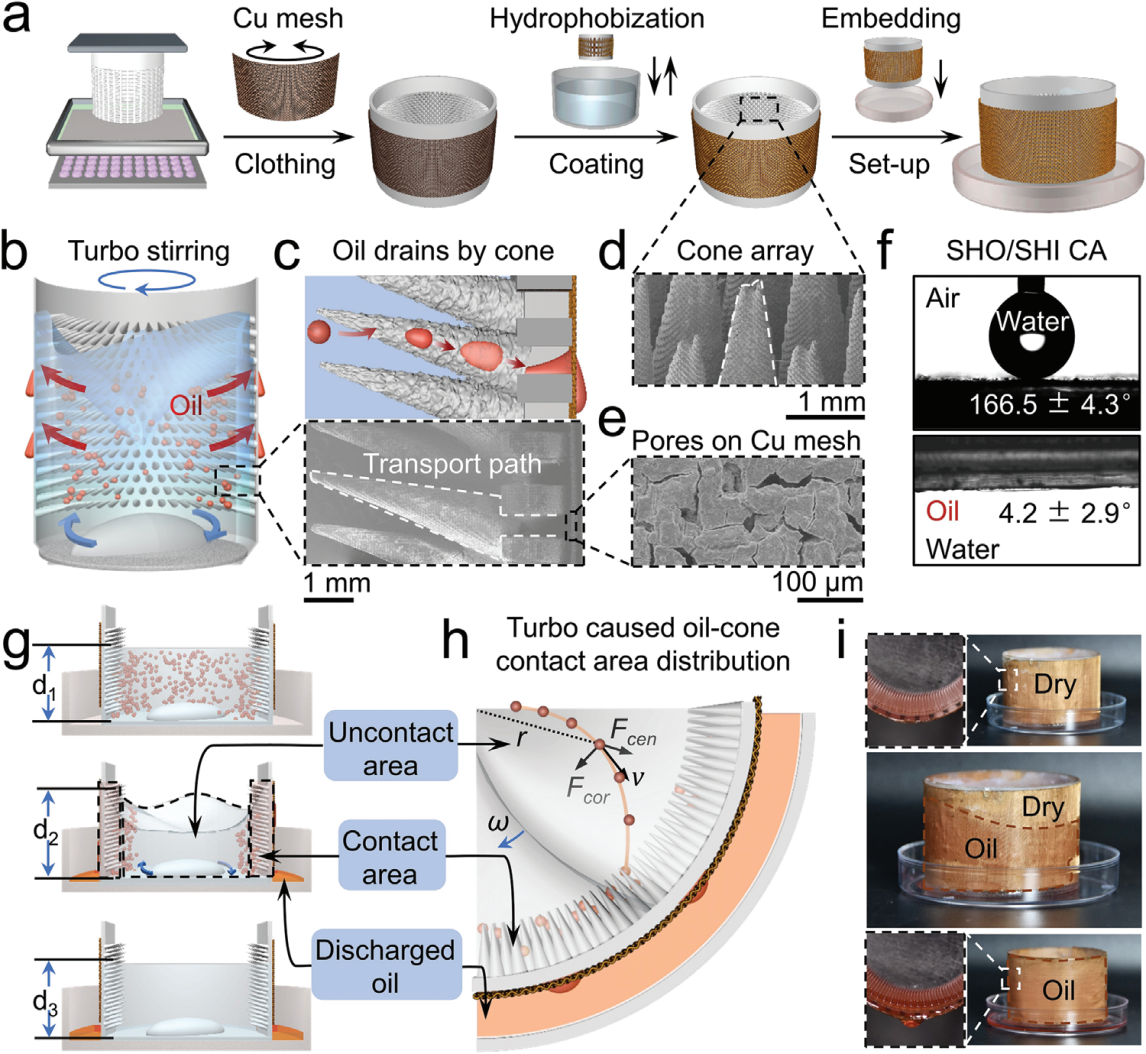
Turbo‐synergistic emulsion separation mechanism based on bio‐inspired cone array barrel (CAB). a) Sketch of the CAB preparation process. The core structure of the CAB is 3D‐printed from light‐cured resin with a piece of Cu mesh clothing the outer wall. After dip‐coated in a hydrophobized solution, the CAB is embedded in a petri dish. b) Schematic of emulsion separation by the CAB under turbo agitation. c) The path of oil droplets capture and transport through the CAB in emulsion (top). SEM image demonstrates the position relationship of cone array, side wall, and Cu mesh (bottom). d) SEM image of tightly arranged cone array with micron concave and convex surface. e) SEM image of Cu mesh after hydrophobization demonstrates distributed micron pores. f) The surface of the CAB showing superhydrophobicity in air (166.5 ± 4.3°) and superoleophilicity underwater (4.2 ± 2.9°). g) Schematic explanation of emulsion separation process in CAB. In the initial state, the liquid level of emulsion in the barrel is d1 (1.64 cm). When turbo agitation of the magneon starts, the liquid level rises to d2 (1.78 cm). The emulsion separation process begins with oil droplets thrown out from uncontact area to contact area approaching the inner wall under centrifugation. The oil droplets are captured by micron structure and subsequently transported though the path on inner wall once contacted with the cone surface. The whole process ends with discharged oil collected in the petri dish while the liquid level of remediated water recovered to d3 (1.62 cm). h) Top‐view schematic of the CAB during emulsion separation process demonstrates turbo‐caused oil‐cone contact area distribution. Under the inertial centrifugal and Coriolis forces, oil droplets near center are thrown onto the cones arrayed on inner wall and drained out afterward. i) Optical images of oil permeating through Cu mesh. Under the gravity, oil permeated from the CAB easily flows down into the petri dish.

Turbo‐synergistic emulsion separation device operates mainly based on a bio‐inspired cone array barrel (CAB), fabricated by 3D‐printing technology with light‐cured resin. The whole preparation process is shown in **Figure** **1**a. There are micron textures on the surface of cones (Figure S1, Supporting Information), as well as holes with the length of 2 mm and the width of 0.5 mm (Figure S2, Supporting Information) on the side wall of 3D‐printed CAB. The water contact angle of 3D‐printed sample is 82.5 ± 2.3° (Figure S3, Supporting Information). A Cu mesh tightly covers the side wall of CAB to reduce the size of 3D‐printed holes. After dip‐coated in a hydrophobized solution, the CAB is embedded in a petri dish to assemble into a liquid‐phase separation device. With the assist of turbo stirring, dispersed oil droplets in emulsion swim toward the side wall under the centrifugal force (Figure 1b). As soon as contacting with a cone, the oil droplet transports spontaneously along the surface to the root of cone and finally drains out through the Cu mesh (Figure 1c, top). Arrayed cone grows on the inner wall at an angle of 70° and the Cu mesh is tightly attached to the outer wall (Figure 1c, bottom), which forms into a transport path for the oil droplet captured by cone. Covered with hydrophobic coating, the micron texture remains on the cone to capture oil (Figure 1d and Movie S1, Supporting Information), while the size of the pore is reduced to increase the hydrostatic pressure of CAB (Figure 1e). As shown in Figure 1f, the surface of a well‐prepared CAB demonstrates superhydrophobicity (SHO) with a contact angle of 166.5 ± 4.3° in air, and superoleophilicity (SOI) with a contact angle of 4.2 ± 2.9° under water. The adhesion force of water in air is 1.9 ± 0.9 µN (Figure S4, Supporting Information). These results reveal that the surface of CAB can capture oil and repel water at the same time. The mechanism of turbo‐synergistic emulsion separation by CAB is shown in Figure 1g. In the initial state, the liquid level of emulsion in the barrel is d1 (1.64 cm). When turbo agitation of a magneton starts, the liquid level rises to d2 (1.78 cm). The emulsion separation process begins with oil droplets thrown out from uncontact area to contact area approaching the inner wall under centrifugal force. Oil droplets are captured by micron textures and subsequently transport though the path on inner wall once contacted with the cone surface. The whole process ends with discharged oil collected in the petri dish while the liquid level of remediated water recovered to d3 (1.62 cm). The difference between d_1_ and d_3_ proves that the oil droplets in emulsion are expelled. Top‐view schematic of partial CAB during emulsion separation process demonstrates turbo caused oil‐cone contact area distribution (Figure 1h). Dispersed oil droplets in emulsion constantly rush from uncontact area to contact area under the action of turbo stirring. Moving oil droplets are captured by cone array in contract area and eventually discharged into the petri dish, meantime the wastewater in CAB is remediated as a result. Figure 1i shows that oil easily penetrates the Cu mesh and flows into the petri dish.

The contact process between oil droplets and micron textures on cone array can be approximated as the coalescence process of two oil droplets. The energy released in the process can be calculated as:^[^ ^28^ ^]^

(1) $$\Pi =E_{\mathrm{surf}}-\Delta p\times V$$

where *E*_surf_ is the increase of surface tension energy, which can be described as:

(2) $$E_{\mathrm{surf}}=\gamma_{OW}\;A=4\pi \gamma_{OW}\;r^{2}=\frac{3\gamma_{OW}V}{r}$$

∆*p* × *V* is the dissipated energy with the change of oil droplet morphology.

(3) $$\Delta p\times V=\frac{2\gamma_{OW}V}{r}$$

Thus, the value of released energy is positive during the process of oil capturing on cone array, which means the dynamic process will occur spontaneously. The key to improve the separation efficiency is to increase the contact between oil droplets and cone surface.

The moving trajectory of an oil droplet in emulsion under turbo stirring is described in detail in Figure 1h. Coriolis effect is a description of the outward motion of a particle in a rotating system. At the stirring speed of *ω*, an oil droplet moves toward the cone array under the resultant force (${\vec{F}}_{\mathrm{res}}$) of Coriolis force and centrifugal force.

(4) $${\vec{F}}_{\mathrm{res}}={\vec{F}}_{\mathrm{cor}}+{\vec{F}}_{\mathrm{cen}}$$

where the Coriolis force (${\vec{F}}_{\mathrm{cor}}$) can be described as:

(5) $${\vec{F}}_{\mathrm{cor}}=2m\vec{\omega }\times \vec{v}$$

and the centrifugal force (${\vec{F}}_{\mathrm{cen}}$) can be described as:

(6) $${\vec{F}}_{\mathrm{cen}}=m\omega^{2}\vec{r}$$

To evaluate the effect of turbo‐synergistic oily wastewater remediation through bio‐inspired CAB, the emulsion separation efficiency can be calculated as:

(7) $$R\left(\%\right)=\left(1-\frac{C_{t}}{C_{0}}\right)\times 100\%$$

where *C*_0_ is the oil content in original emulsion and *C_t_* is the oil content in liquid remained in CAB after separation, measured by Infrared Spectrophotometer.

A straightforward result of turbo‐synergistic oil–water mixture separation through CAB is shown in Figure S5 (Supporting Information). Comparing the oil–water mixture in CAB with and without turbo stirring, the time taken for separation varies greatly. It takes 30 s with turbo stirring, while 120 s without stirring to see obvious separation result (Figure S6, Movies S2 and S3, Supporting Information). With the synergy of turbo stirring, floated oil is dispersed in water as small droplets so that more arrayed cones on inner wall work in the separation process (Figure S7, Supporting Information), resulting in a shortened time of separation. For different types of oil–water mixture, the separation efficiencies maintain above 99.9% under turbo stirring in bio‐inspired CAB (Figure S8, Supporting Information). Since the turbo‐synergistic CAB is aimed to deal with wastewater, emulsified oil is prepared to compare the separation effect with and without turbo stirring (Figure S9, Supporting Information). Compared with the experiment in CAB without stirring, emulsion in the CAB with stirring turns transparent significantly and meanwhile oil drains out into the petri dish from the barrel in 10 min, which demonstrates the good synergistic effect of turbo stirring with gradient cone structure.

**Figure 2:**
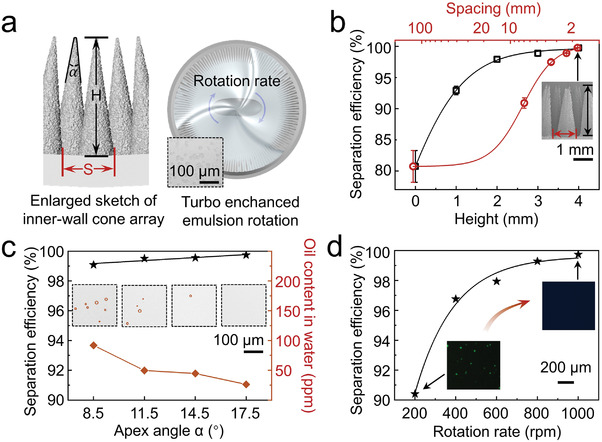
Device parameters affecting the separation efficiency. a) Schematic diagrams of variable control. The height (H) and spacing (S) of the cone array, the angle of cone apex (*α*) and rotation rate (R) are variable. b) Separation efficiencies of CAB with different heights and spacings of the cone array. Larger height and smaller spacing contribute to higher separation efficiency. c) Separation efficiencies of CAB with different apex angles of the cone. Higher separation efficiency benefit from larger angle of cone apex. d) Separation efficiencies of CAB with different rotation rates.

To develop the best separation effect of well‐prepared CAB, several device parameters including the height of cone (H), the spacing between two adjacent cones (S), the angle of cone apex (*α*), and the rotation rate (R) are discussed in **Figure** **2**. Figure 2a demonstrates schematic diagrams of variable control. A series of CAB devices with different heights and spacings of cone are designed and fabricated, while the heights vary from 0 to 4 mm and the spacings vary from 1.7 to 125 mm. Optical microscope images of emulsion before and after separation by different CAB are shown in Figures S10 and S11 (Supporting Information). As shown in Figure 2b, the CAB with higher height and smaller spacing provides more area for oil droplets to be captured, thus, the separation efficiency improves with increasing height or decreasing spacing. When the cone array is fabricated with a height of 4 mm and a spacing of 1.7 mm, the separation efficiency reaches 99.7% (Movie S4, Supporting Information).

Furthermore, the apex angle of cone also affects the separation efficiency of CAB (Figure 2c). When the apex angle of cone varies from 8.5° to 17.5° (Figure S12, Supporting Information), the separation efficiency improves from 99.0% to 99.7%. In the case of the CAB with same height and spacing, the CAB with larger apex angle of cone exposes larger contact area, which results in more effective capture of oil droplets. Meanwhile, larger apex angle of cone provides a greater *F_L_*, which enables faster transport of captured oil along the cone. Therefore, the separation efficiency improves as the apex angle increases. When the apex angle of cone is 17.5°, there is no obvious oil droplets in the microscope image of emulsion after separation (Figure 2c, insets).

In addition to the structure of CAB, the rotation rate during the process is vital for emulsion separation. As shown in Figure S13 (Supporting Information), the separation efficiency increases with the extension of separation time under the speed of 200 and 1000 rpm. After 10 min, the change of separation efficiency is gentle, revealing that the separation process is basically completed in 10 min. The separation efficiency at 10 min under different rotation rate is shown in Figure 2d. The CAB preforms better separation efficiency with the larger rotation rate. When the rotation rate approaches 1000 rpm, the separation efficiency reaches 99.7%. The optical microscope images of emulsion over time under 1000 rpm are shown in Figure S14 (Supporting Information), demonstrating the oil droplets decreasing gradually and disappearing finally at 10 min. The faster the CAB rotates, the greater the probability of effective capture of small oil droplets happens.

**Figure 3:**
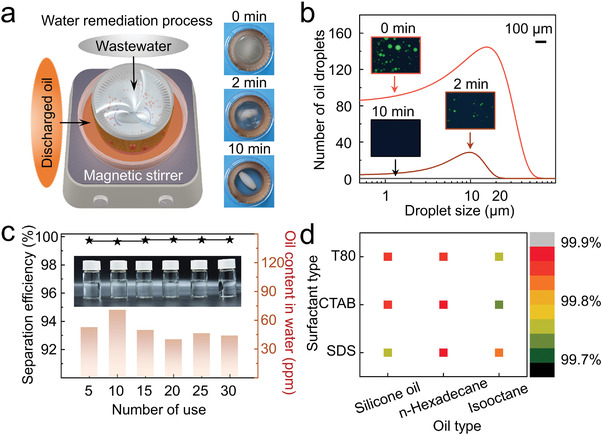
Stability and applicability tests of CAB in emulsion separation. a) Schematic diagram and video snapshoots of the separation experiment. After stirring for 10 min, the cloudy emulsion becomes clear. b) Particle size statistics and fluorescent images (insets) of emulsion before separation, after 2 and 10 min. With the increase of separation time, the average size and number of oil droplets in the emulsion decrease. After 10 min, there is no obvious oil droplets in view. c) In consecutive 30 times of oily wastewater remediation, the separation efficiencies of CAB keep above 99.5%. d) CAB shows high efficiencies in separating emulsion with different types of surfactant and oil.

Schematic diagram of oily wastewater remediation process and screenshots of separation process are shown in **Figure** **3**a. Oily wastewater in CAB is turbo‐synergistically disposed under magnetic stirrer and the captured oil permeates through CAB wall and is discharged in a petri dish. Cloudy emulsion gradually becomes clear in 10 min. The size statistics of oil droplets in emulsion as the process goes on are shown in Figure 3b. Average size and number of oil droplets in emulsion decreases over time. Fluorescence microscope images of emulsion are corresponding to the size statistics of oil droplets at different separation time (Figure 3b, insets). At 10 min, no oil droplets are observed in the emulsion.

In consecutive 30 times of oily wastewater remediation, the separation efficiencies of the same CAB remain above 99.5%, which reveals that the structure of CAB stays very stable, and maintains not damaged under the synergy of turbo rotation (Figure 3c). Except for the test of stability, the applicability result of CAB for different emulsions is shown in Figure 3d. The comparison images of emulsion before and after separation are shown in Figure S15 (Supporting Information), which different surfactants (sodium dodecyl sulfate (SDS), hexadecyl trimethyl ammonium bromide (CTAB), and Tween 80 (T80)) and oil (silicone oil, *n*‐hexadecane, and isooctane) are chosen to form into different emulsions. No oil droplets are observed in the view of each emulsion after separation and the separation efficiencies for different emulsion keep above 99.7%, which proves that the turbo‐synergistic remediation method in bio‐inspired CAB is effective for most kinds of emulsions. To test the durability of the as‐prepared cone array barrel, we conducted emulsion separation experiments in extreme acid and alkaline solutions, as well as in UV irradiation. Sulfuric acid solution is used instead of water to prepare the emulsion of pH 2 with the existence of surfactant CTAB, while sodium hydroxide solution is used instead of water to prepare emulsion of pH 12 with the surfactant SDS. The separation efficiency results are demonstrated in Figure S16 (Supporting Information). Turbo‐synergistic CAB maintains a high separation efficiency of better than 90% under acid/alkaline solutions or 7‐day UV irradiation. However, the separation effect is still decreased in some degree since the light curing resin material in 3D‐printed barrel cannot tolerate the acid and alkali for a long‐time use. Besides, a friction resistance test is achieved with tape peeling up to ten times to a cone array device and resulted in a good separation effect (Figure S17, Supporting Information).

**Figure 4:**
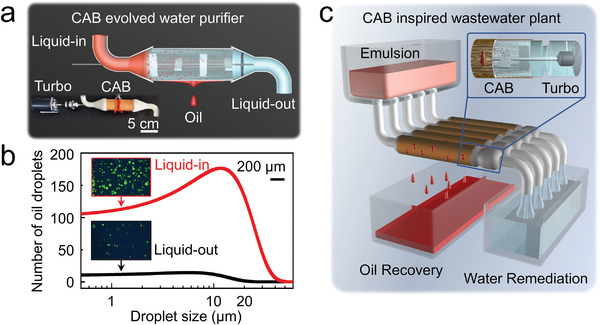
Future technology design of water purification based on the CAB. a) The optical image and perspective diagram of a made‐in‐lab CAB‐evolved water purifier. Stirred by a turbo, oily wastewater flows through a structure‐improved CAB at 10 mL min ^−1^, with purified water left while oil drains out from CAB wall. b) Particle size statistics of oil droplets in liquid before and after separation by the evolved water purifier. The amount of oil droplets in emulsion decreases significantly shown in insets, the fluorescence microscope images of liquid‐in and liquid‐out. c) The blueprint of future industrial wastewater treatment plant based on turbo‐synergistic CAB.

To apply the turbo‐synergistic water remediation strategy through CAB in daily and industrial scenes, we attempt preliminary designs to achieve continuous emulsion separation. Hydraulic resistance (Figure S18, Supporting Information) and pure oil flux (Figure S19, Supporting Information) of the cone array barrel are first tested to support the design of future applications. Based on our strategy, a CAB‐evolved water purifier is prepared in **Figure** **4**a, with a length of 10 cm, a diameter of 4 cm, and cone array on its side wall equips emulsion inlet and outlet. The magnetic stirrer is replaced by a mechanical motor to provide turbo synergy and the emulsion flows into the device at 10 mL min^−1^. The statistics of oil droplets and microscopic images of emulsion liquid‐in and liquid‐out of the device are shown in Figure 4b, demonstrating that the number of oil droplets varies significantly in two images and the average size of oil droplets after separation is much smaller than that before separation. All these results reveal the effectiveness of the device for emulsion separation. In our further assumption, by optimizing the project, a turbo‐synergistic CAB‐inspired wastewater plant will be built to remediate surfactant‐stabilized oil–water mixture, and recover oil resource at the same time (Figure 4c).

Turbo‐synergistic separation of oil–water mixture is achieved by dispersing oil phase into water phase to form into tiny droplets with the dynamic liquid motion, which increases the contact area between oil and the side wall. When the water and oil are replaced by original solution (*L*_1_) and extractant (*L*_2_), extraction takes place in CAB (Figure S20, top, Supporting Information). The extraction process is shown in Figure S20, bottom (Supporting Information). The silicone oil (*L*_2_) is used to extract iodine in KI solution (*L*_1_). In the process of turbo‐synergistic motion in CAB, the silicone oil with iodine ($L_{2}^{\prime }$) penetrates through the side wall of CAB by wettability and the KI solution ($L_{1}^{\prime }$) remains (Movie S5, Supporting Information). The color of silicone oil and KI solution change obviously before and after extraction. The content of iodine in KI solution after extraction is determined as 2.8 mg L^−1^ by a UV–vis absorption spectrum. Compared with 200 mg L^−1^ in original solution, the extraction efficiency reaches 98.6% (Figure S21, Supporting Information). This strategy of turbo‐synergistic liquid remediation in bio‐inspired CAB not only works in environmental case like oil–water separation but also provides a dynamic design idea in processes of any industrial and pharmaceutical field involving liquid phase separation.

## 3 Conclusion

With the inspiration from nature, we find gradient cone structure and wettability difference to achieve efficient oil–water separation, and taking it one step further we develop turbo‐synergistic oily wastewater remediation with man‐made dynamic power. Under the centrifugal force in cone array barrel, oil droplets in emulsion are captured and then penetrated out along the superhydrophobic/superoleophilic path on cones, with only remediated water remained. Turbo‐synergistic CAB is robust and common for most kinds of surfactant‐stabilized oil–water emulsion, and reaches a separation efficiency of 99.7%. By modifying the structure, the CAB could evolve into practical devices in continuous daily and industrial processes. This strategy has also been proved to be effective in organic extraction, and is expected to work in more liquid mixture‐handling scenarios in the field of environmental treatment, resource recovery, and chemical manufacture.

## 4 Experimental Section

### Materials

Copper mesh (500#, Shanghai Huadong Composite Insulation Filter Cloth Screen Factory), 3D‐printing UV‐sensitive resin (clear, Anycubic), ultra‐ever dry (top coat 4001, Ultratech international, Inc.), dimethylsilicone oil (PMX200‐5CS, Dow Corning), isooctane (Aladdin), *n*‐hexadecane (Aladdin), Tween 80 (Aladdin), sodium dodecyl sulfate (Aladdin), hexadecyl trimethyl ammonium bromide (Aladdin), CCl_4_ (Tianji Aoran Fine chemical research Institute), I_2_ (Aladdin), and KI (Aladdin) were purchased as described.

### Instrument and Characterization

Scanning electron microscopy (SEM) images were obtained by a Field emission scanning electron microscope (HITACHI Su8010, Japan). Oil content in filtrate was measured by Infrared Spectrophotometer OIL 480. Contact angles were measured by a contact angle system (Data‐physics OCA20, Germany). Liquid–solid adhesion force was measured by a dynamic contact angle machine (Data Physics DCA 20, Germany). Optical and fluorescent microscopy photographs were snapped by a microscope (Olympus BX51 with DP80, Japan). Digital photos and videos were caught by a smartphone (Huawei Mate 30, China). The process of capturing oil droplets by a single cone was recorded with the fluorescent microscopy (Olympus BX51 with DP80, Japan).

### Preparation of the Microstructured Cone Array Barrel

The 3D‐printing machine was Anycubic Mono X. The copper mesh was attached to the outer wall of the 3D‐printed sample using UV‐sensitive resin under UV light. After that, the ultra‐ever dry solution was coated on the surface of the cone array barrel.

### Preparation of the O/W Emulsion

The ratio of oil, water, and surfactant was 4 mL: 196 mL: 40 mg; and the emulsion was prepared by mechanical stirring at 550 rpm for 6 h. The types of oils were dimethylsilicone oil, isooctane, and *n*‐hexadecane, while the types of surfactants are Tween 80 (T 80), sodium dodecyl sulfate (SDS), and hexadecyl trimethyl ammonium bromide (CTAB).

### Oil–Water Separation Process

Oil (5 mL) and water (15 mL) mixture was added into the CAB and stirred with a magneton for 30 s. The types of oils were silicone oil, isooctane, and *n*‐hexadecane.

### Emulsion Separation Process

A total of 15 mL emulsion was added into the CAB and stirred with a magneton for 10 min. The captured oil penetrated out from the side wall of the CAB and drained into the petri dish.

### Determination of Oil Content in Water After Separation

A total of 20 mL CCl_4_ was first added into a beaker to extract 10 mL remediated water after separation, and then detected by Infrared Spectrophotometer OIL 480.

### Extraction Process

The I_2_ content in 0.1 mol L^−1^ KI solution was 200 mg L ^−1^. A total of 5 mL solution was stirred in the CAB at 1000 rpm. A total of 15 mL silicone oil was slowly dripped into the CAB. The silicone oil containing I_2_ penetrated from the CAB. The extraction process took 2 min.

## Conflict of Interest

The authors declare no conflict of interest.

## Supporting information

Supporting Information

Supplemental Movie 1

Supplemental Movie 2

Supplemental Movie 3

Supplemental Movie 4

Supplemental Movie 5

## Data Availability

The data that support the findings of this study are available in the supplementary material of this article.
