# Supplementary material for: Turbo‐Synergistic Oily Wastewater Remediation in Bio‐Inspired Cone Array Barrel
Source: Adv Sci (Weinh). 2022 Oct 6;9(33):2204244. doi: 10.1002/advs.202204244 (PMC9685448; doi:10.1002/advs.202204244)
Supplement: Supplementary file 1 — Supporting Information [file ADVS-9-2204244-s006.pdf]

ADVANCED SCIENCE
Open Access

Supporting Information

for *Adv. Sci.*, DOI 10.1002/advs.202204244

Turbo-Synergistic Oily Wastewater Remediation in Bio-Inspired Cone Array Barrel

*Bing Wang, Xianfeng Luo, Yawei Feng, Linfeng Yang, Chunhui Zhang, Zhichao Dong, Lei Jiang and Haoyu Dai**

Supporting Information

Turbo Synergistic Oily Wastewater Remediation in Bio-inspired Cone Array Barrel

*Bing Wang, Xianfeng Luo, Yawei Feng, Linfeng Yang, Chunhui Zhang, Zhichao Dong, Lei Jiang, and Haoyu Dai**

Supporting information includes:

One PDF file with Supporting Figures. Also with captions of Supporting Figures and Supporting Movies. Five Supporting Movie video files (mp4.).

Figure S1. SEM images of 3D printed cone array with micron texture.

Figure S2. SEM image of 3D printed side wall with holes.

Figure S3. Contact angle characterization of 3D printed surface.

Figure S4. Adhesion force test to water droplets on the surface after hydrophobization.

Figure S5. Separation of oil-water mixture and microscopic image of water phase after separation.

Figure S6. Comparison of separation time with and without turbo stirring.

Figure S7. Comparison of the states of oil and water with and without turbo stirring.

Figure S8. Separation efficiency of different oil-water mixture by CAB under turbo stirring.

Figure S9. Comparison of emulsion separation effect through the CAB with and without stirring.

Figure S10. Comparison of separation effects by the CAB with different cone heights.

Figure S11. Comparison of separation effects by the CAB with different cone spacings.

Figure S12. SEM images of cone array with different apex angles.

Figure S13. Comparison of separation efficiencies by the CAB under 200 rpm and 1000 rpm stirring.

Figure S14. The separation effect and oil content in water of different separation time by the CAB under 1000 rpm stirring.

Figure S15. Images of emulsion with different surfactant/oil types before and after separation.

Figure S16. Emulsion separation efficiency test in extreme acid/alkaline solutions and UV irradiation.

Figure S17. Friction resistance test of the cone array device.

Figure S18. Hydraulic resistance of the cone array barrel.

Figure S19. Pure oil flux of the cone array barrel.

Figure S20. Turbo synergistic extraction process of Iodine in the CAB.

Figure S21. Determination of I_2 content by UV-vis absorbance spectrum.

Movie S1. Oil droplets captured on a cone. Tiny oil droplets being captured by a 3D-printed cone can be observed when the emulsion is sprayed toward the cone.

Movie S2. Oil-water mixture separation in the CAB without stirring. 15 mL oil-water mixture is fully separated in the CAB without stirring in 120 seconds.

Movie S3. Oil-water mixture separation in the CAB with stirring. 15 mL oil-water mixture is fully separated in the CAB with stirring in 30 seconds.

Movie S4. Turbo synergistic emulsion separation experiment in the CAB. After 10 minutes separation of 15 mL emulsion in CAB with stirring, remediated water in the CAB turns transparent while oil penetrates out into the petri dish.

Movie S5. Turbo synergistic extraction process of Iodine in the CAB. While adding oil into the CAB, oil containing I_2 is captured by the cone array and penetrates out from the barrel under turbo stirring. After the process, I_2 is enriched from 5 mL KI solution to silicone oil.

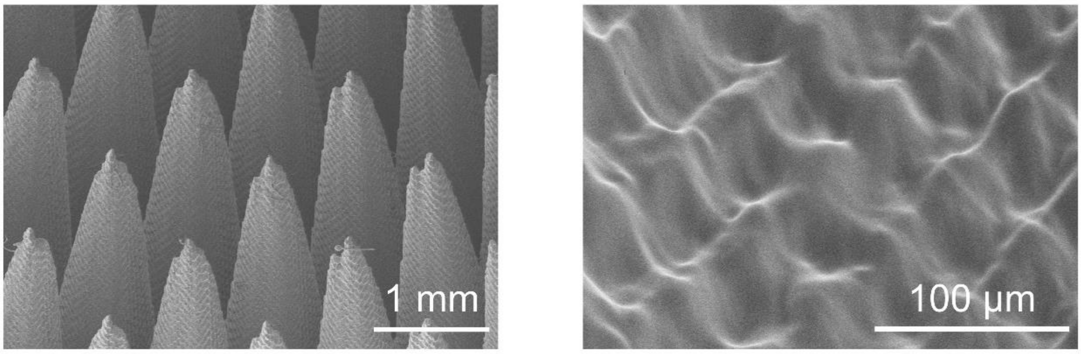

Figure S1. SEM images of 3D printed cone array with micron texture. The morphology of 3D printed cone array is regular. In the magnification of SEM image, the surface of cone demonstrates micron texture, which increases the surface roughness.

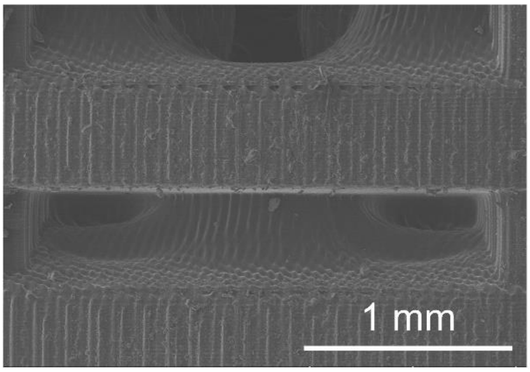

Figure S2. SEM image of 3D printed side wall with holes. The holes with the width of 2 mm and the height of 0.5 mm are designed for oil to penetrate out.

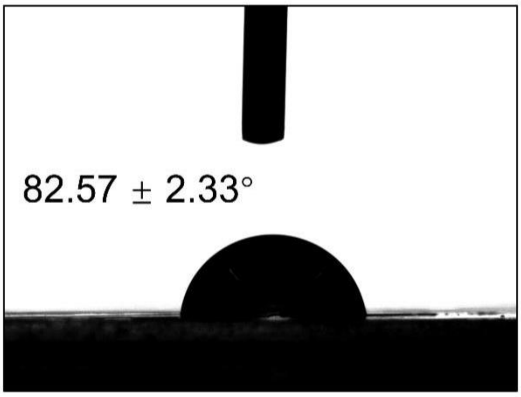

Figure S3. Contact angle characterization of 3D printed surface. The water contact angle of surface in air is 82.5 ± 2.3° .

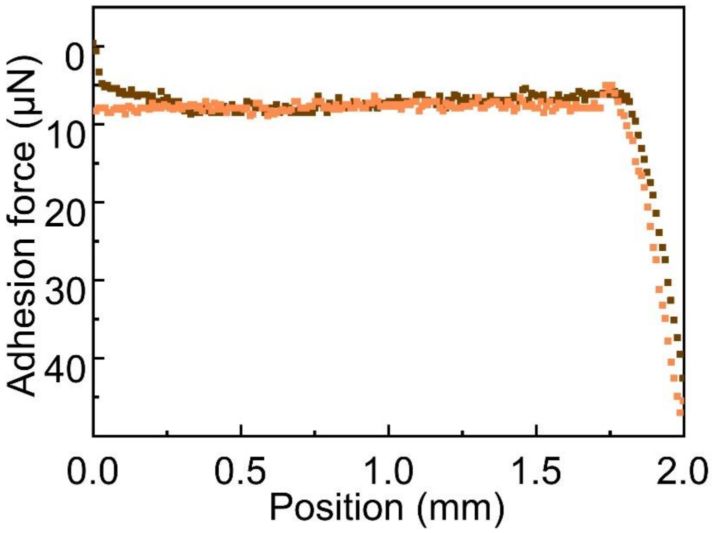

Figure S4. Adhesion force test to water droplets on the surface after hydrophobization. The adhesion force of water to the modified surface is 1.9 ± 0.9 μN.

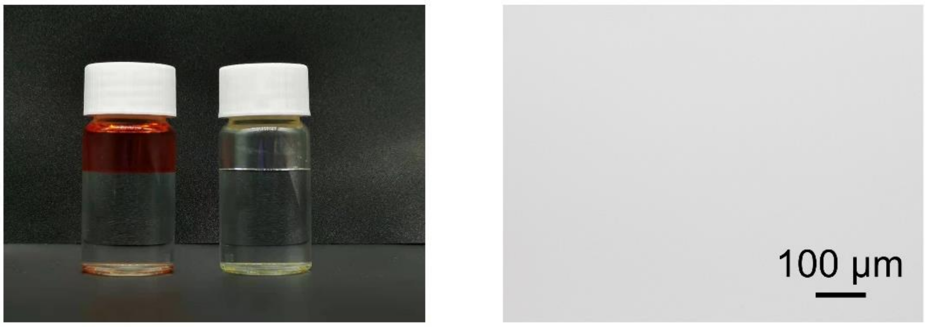

Figure S5. Separation of oil-water mixture and microscopic image of water phase after separation. After separation, there is no obvious oil in the view.

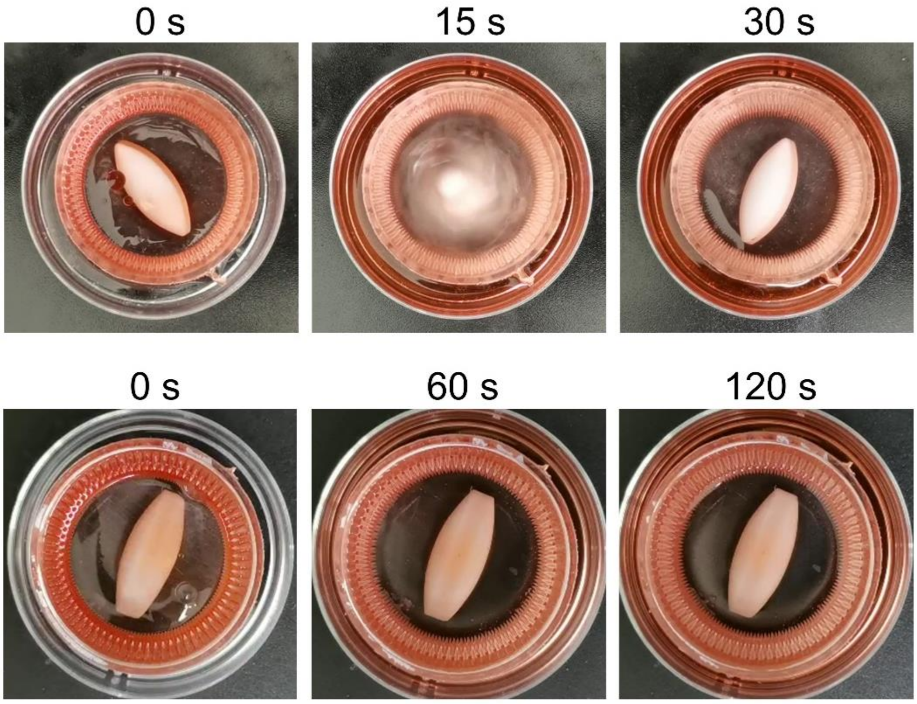

Figure S6. Comparison of separation time with and without turbo stirring. Under stirring, the separation time can be shortened and the separation effect can be improved.

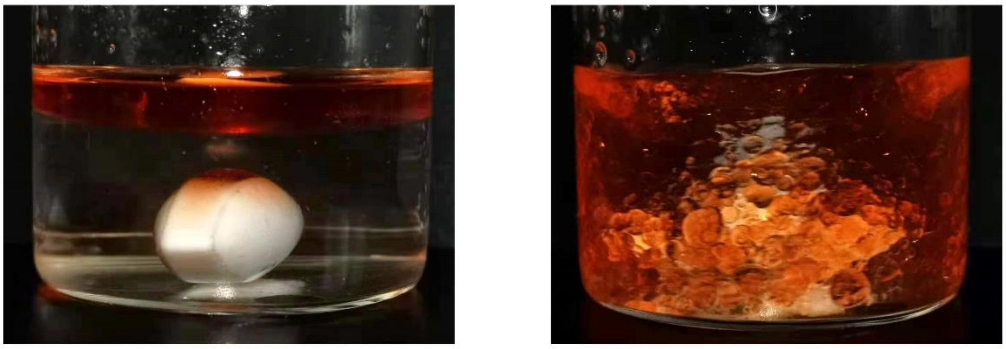

Figure S7. Comparison of the states of oil and water with and without turbo stirring.

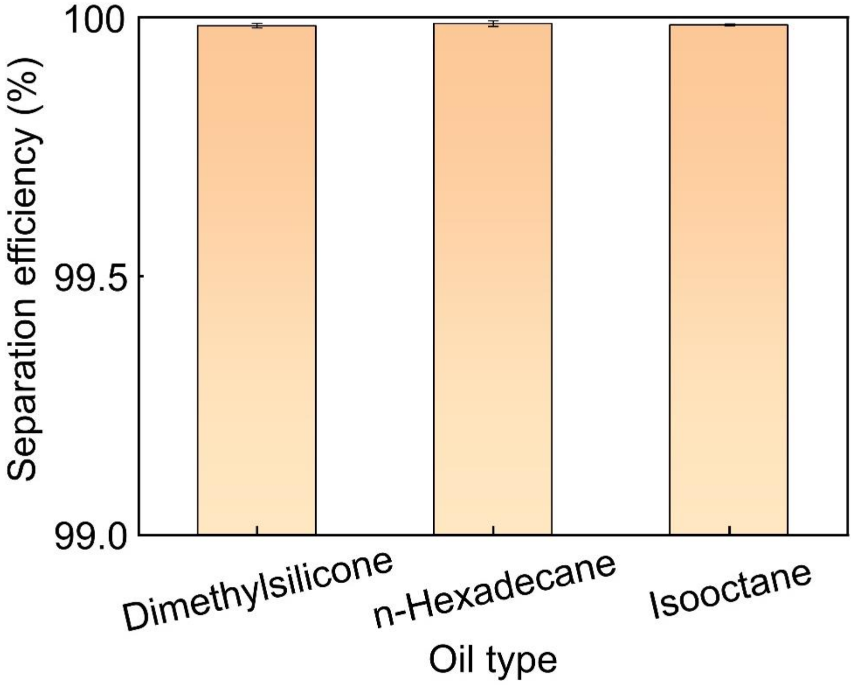

Figure S8. Separation efficiency of different oil-water mixture by CAB under turbo stirring.

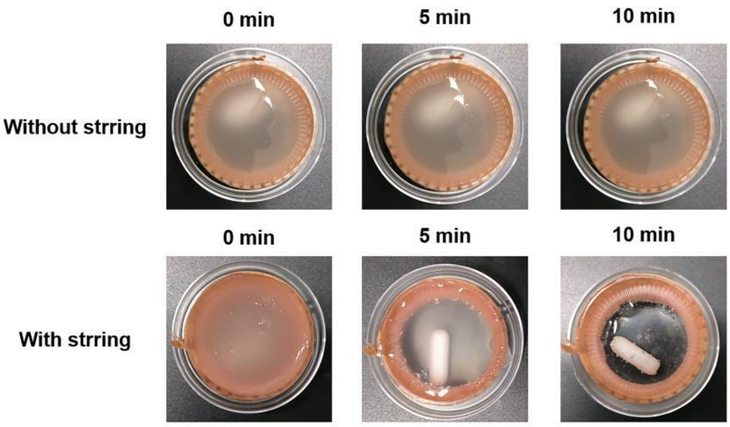

Figure S9. Comparison of emulsion separation effect through the CAB with and without stirring. Compared with the experiment in CAB without stirring, emulsion in the CAB with stirring turns transparent significantly and meanwhile oil drains out into the petri dish from the barrel in 10 minutes.

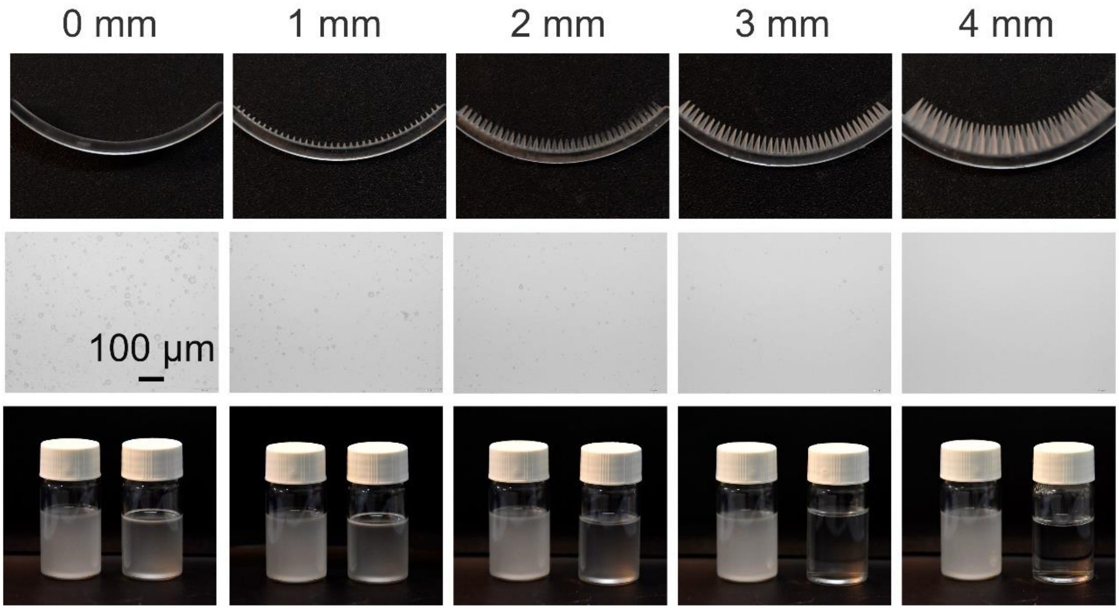

Figure S10. Comparison of separation effects by the CAB with different cone heights. With the larger height, the cone array can provide more surface area for capturing oil droplets.

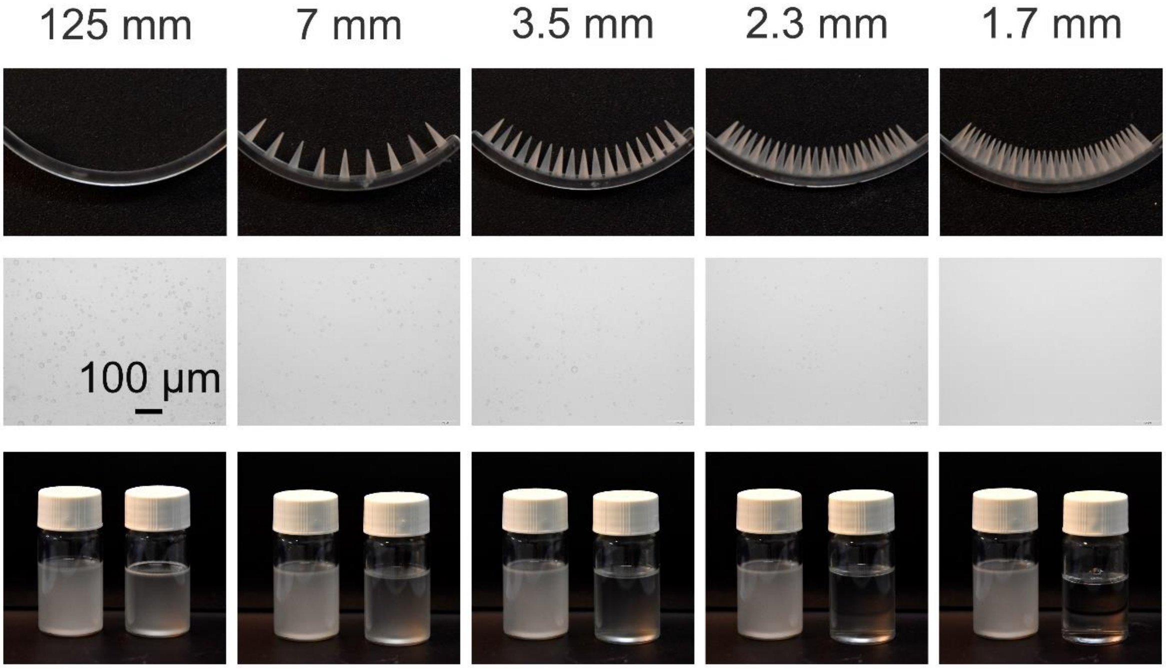

Figure S11. Comparison of separation effects by the CAB with different cone spacings. With the smaller spacing, the cone array has a larger number of cones, which can provide more area for capturing oil droplets.

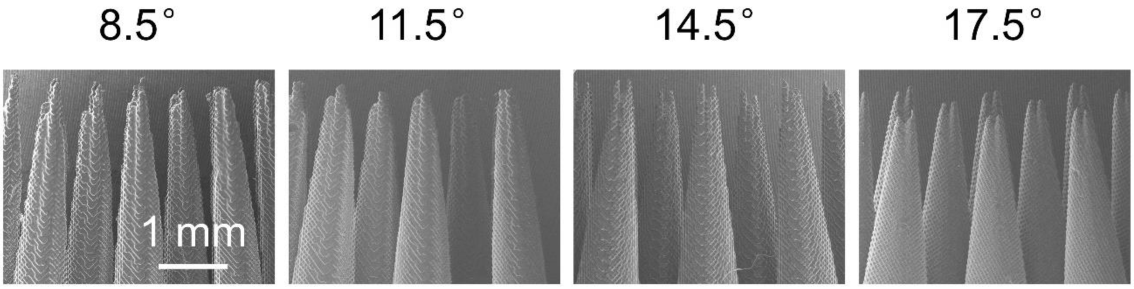

Figure S12. SEM images of cone array with different apex angles. At the same height of the cone array, the larger the apex angle is, the larger area the cone array can provide.

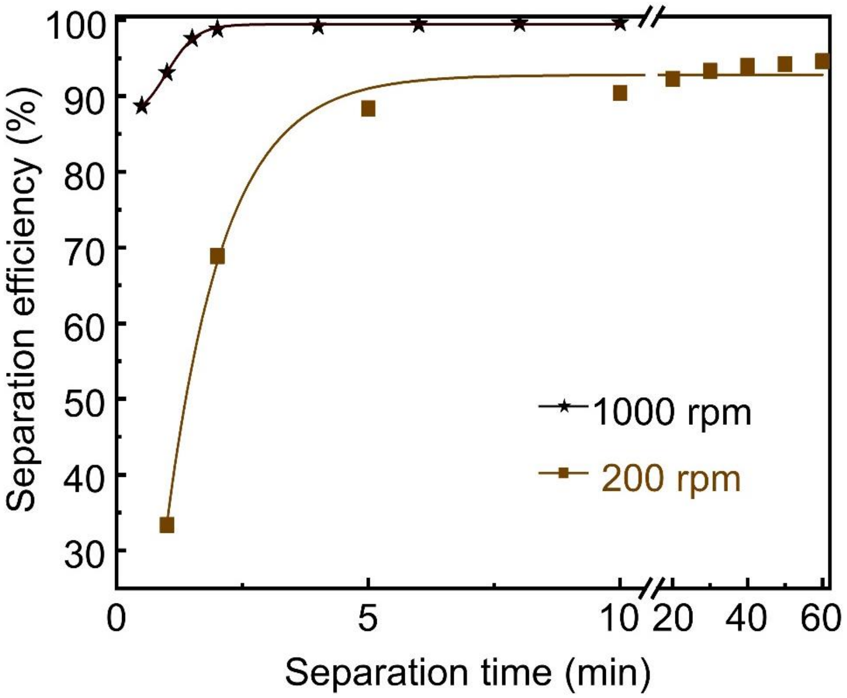

Figure S13. Comparison of separation efficiencies by the CAB under 200 rpm and 1000 rpm stirring. The separation efficiency can be improved by prolonging the separation time. Under a higher speed, the separation time is reduced.

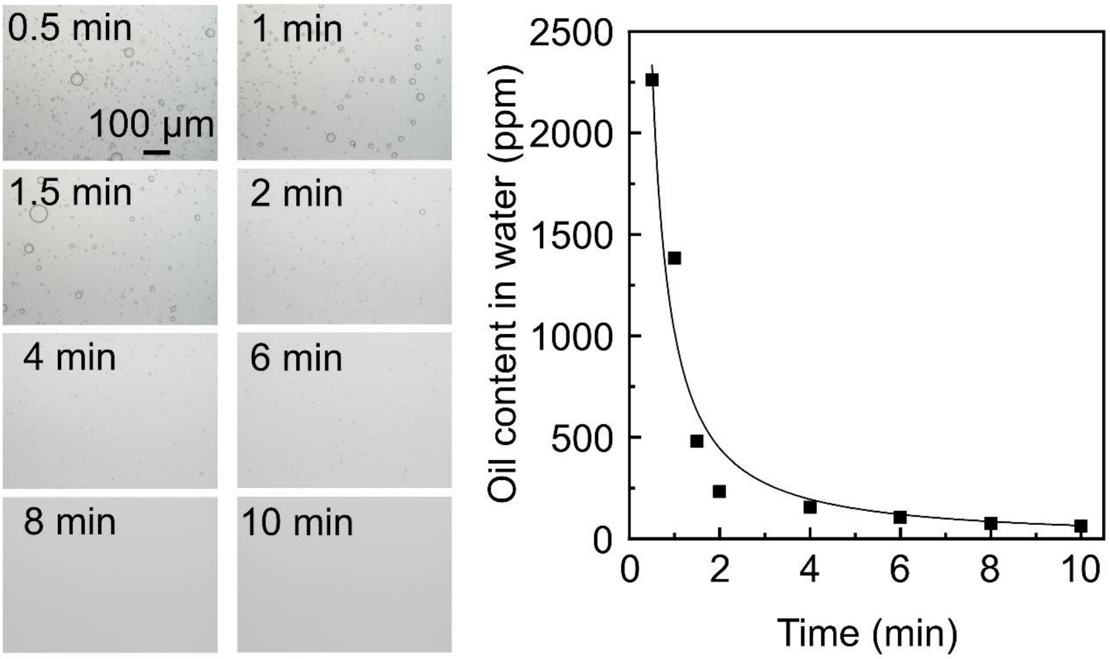

Figure S14. The separation effect and oil content in water of different separation time by the CAB under 1000 rpm stirring. With the extension of separation time, the oil content in water decreases rapidly.

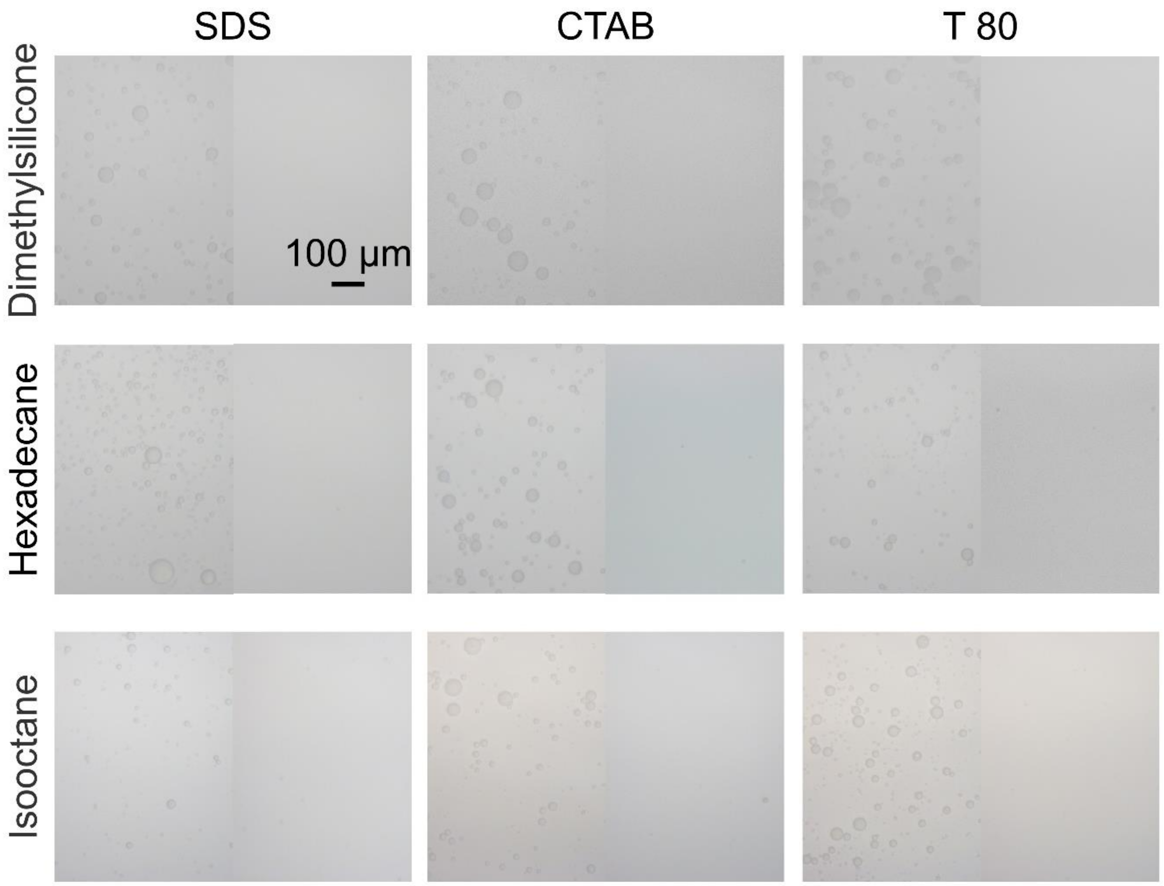

Figure S15. Images of emulsion with different surfactant/oil types before and after separation. After separation, almost no oil droplets are observed in the view.

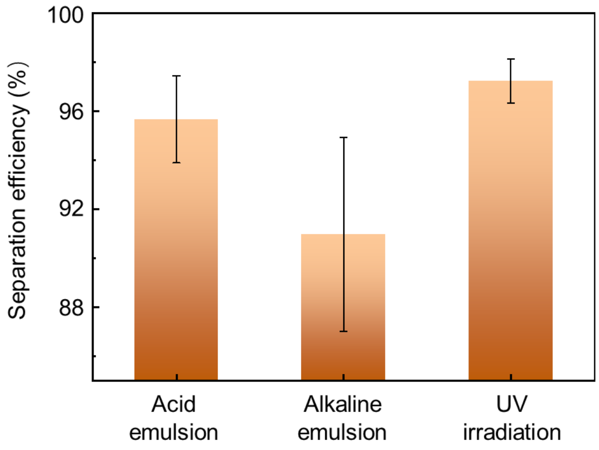

Figure S16. Emulsion separation efficiency test in extreme acid/alkaline solutions and UV irradiation. The turbo synergistic CAB maintains a high separation efficiency of better than 90% under acid/alkaline solutions or 7-day UV irradiation.

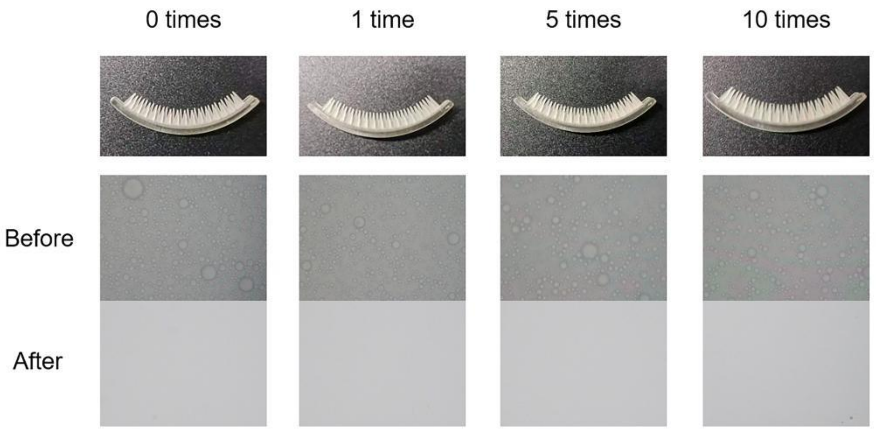

Figure S17. Friction resistance test of the cone array device. The as-prepared device results in a good separation effect within 10 times of tape peeling.

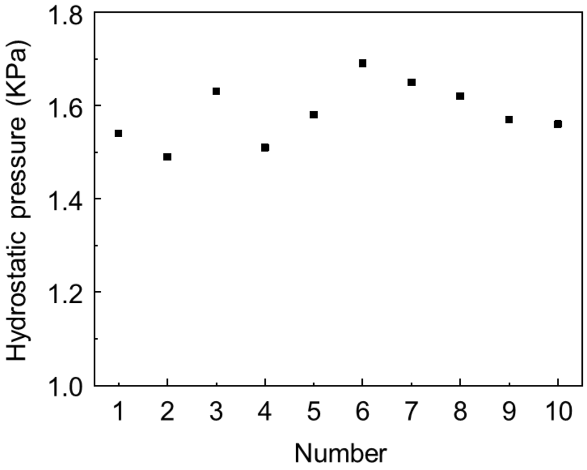

Figure S18. Hydraulic resistance of the cone array barrel. The hydraulic pressure in the CAB keeps about 1.6 KPa in continuous 10 times of tests.

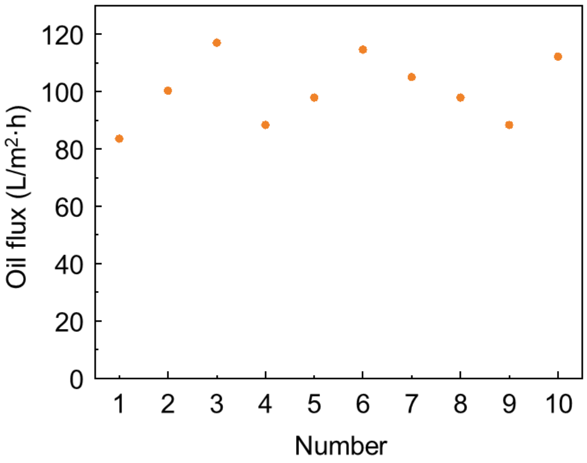

Figure S19. Pure oil flux of the cone array barrel. The oil flux remains about 100 L/m^{2}· h.

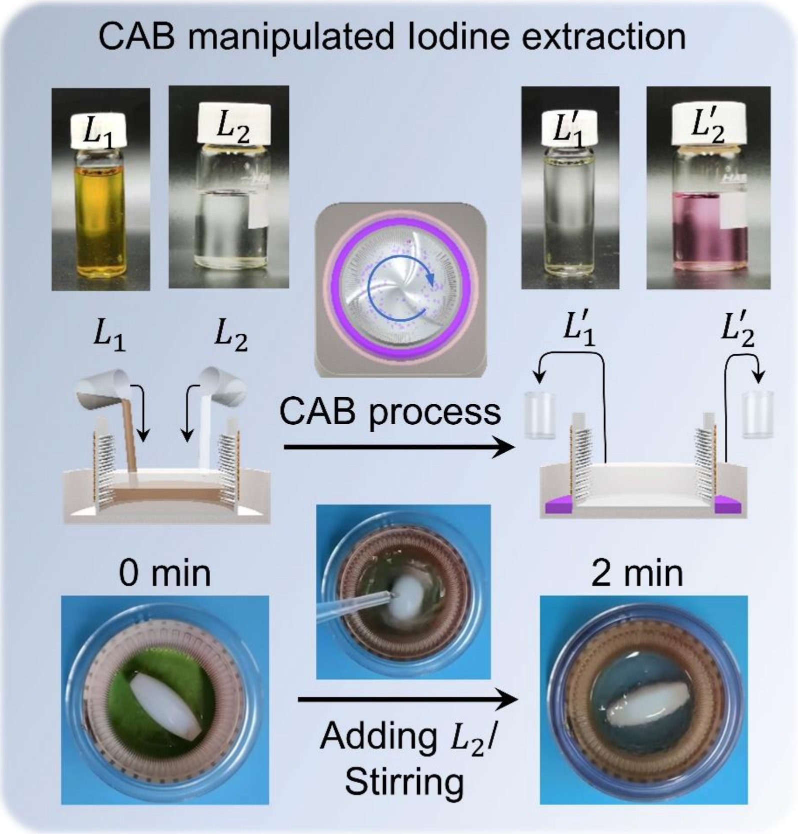

Figure S20. Turbo synergistic extraction process of Iodine in the CAB. Under turbo stirring, I_2 is enriched from KI solution to silicone oil. Oil containing I_2 is captured by the cone array and removed from the barrel.

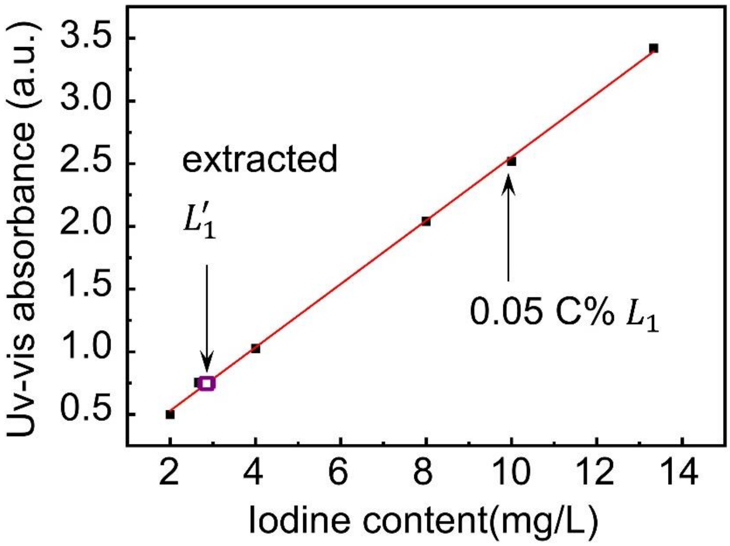

Figure S21. Determination of I_2 content by UV-vis absorbance spectrum. The I_2 concentration in L_1' is 1.4% of that in L_1.
